# Digital therapeutics in the hospital for suicide crisis – content and design recommendations from young people and hospital staff

**DOI:** 10.1177/20552076241230072

**Published:** 2024-02-14

**Authors:** Demee Rheinberger, Rachel Baffsky, Lauren McGillivray, Daniel Z Q Gan, Mark Larsen, Michelle Torok

**Affiliations:** 1 102934 Black Dog Institute, 7800 University of New South Wales, Sydney, Australia; 2 Orygen, Parkville, Australia; 3 Centre for Youth Mental Health, The University of Melbourne, Australia

**Keywords:** Suicide, eHealth, apps, young people, hospital, hospital staff, qualitative

## Abstract

**Objective:**

Hospital emergency departments lack the resources to adequately support young people who present for suicidal crisis. Digital therapeutics could fill this service gap by providing psychological support without creating additional burden on hospital staff. However, existing research on what is needed for successful integration of digital therapeutics in hospital settings is scant. Thus, this study sought to identify key considerations for implementing digital therapeutics to manage acute suicidal distress in hospitals.

**Method:**

Participants were 17 young people who recently presented at the hospital for suicide-related crisis, and 12 hospital staff who regularly interacted with young people experiencing mental ill-health in their day-to-day work. Interviews were conducted via videoconference. Framework analysis and reflexive thematic analysis were used to interpret the data obtained.

**Results:**

Qualitative insights were centred around three major themes: hospital-specific content, therapeutic content, and usability. Digital therapeutics were seen as a useful means for facilitating hospital-based assessment and treatment planning, and for conducting post-discharge check-ins. Therapeutic content should be focused on helping young people self-manage suicide-related distress while they wait for in-person services. Features to promote usability, such as the availability of customisable features and the use of inclusive design or language, should be considered in the design of digital therapeutics.

**Conclusions:**

Digital therapeutics in hospital settings need to benefit both patients and staff. Given the unique context of the hospital setting and acute nature of suicidal distress, creating specialty digital therapeutics may be more viable than integrating existing ones.

## Introduction

Over the last two decades, the rates of self-harm and suicide in Australian young people have steadily increased.^1^ While there is strong evidence for the effectiveness of behavioural therapeutic modalities (e.g. cognitive behavioural therapy and dialectical behavioural therapy) in reducing suicidal thoughts and behaviours,^2^ there are significant structural barriers to accessing treatment.^3^ Even when young people do access face-to-face treatment, 39% will not disclose their suicidal thoughts to their mental health professional.^4^ As such, there is increasing need for novel models of care that increase young people's access to suicide prevention support in ways that they feel comfortable and willing to engage with.

Technology solutions are now recognised as an important component of healthcare for chronic, modifiable conditions, such as mental health.^5^ Research shows that many young people prefer to engage with mental health support and treatment digitally rather than in-person.^6^ Meta-analytic evidence shows that digital therapeutics (treatment delivered via smartphones, tablets, or computers) can significantly reduce symptom severity of suicidal thoughts and behaviours, and pooled evidence suggests that age does not moderate these effects.^7–9^ While there are currently few trials of youth-direct digital therapeutics that target suicidal thoughts and behaviours, there is promising evidence that these tools can benefit young people. For example, a recent Australian study showed in a randomised controlled trial that a self-guided dialectical behavioural therapy-based app significantly reduced suicidal ideation.^10^ While many digital mental health tools can already be publicly accessed via app stores,^11^ including some developed by researchers, progress to integrate these tools into healthcare settings, as part of routine clinical care, remains slow.

Hospitals are an implementation setting of particular interest for integrating digital suicide prevention into existing models of care. Hospitals are often utilised in the height of suicidal distress (suicidal thoughts, plans, or attempts) by young people to access lifesaving medical intervention and psychological support. Young people who present to hospital in suicidal distress also have a considerably higher risk of suicide compared to youth who self-harm and experience ideation in the community.^12^ Despite the important role hospitals have in suicide prevention, young people often report not receiving adequate care for their suicidal distress when attending an emergency department.^13,14^ There is evidence that a negative hospital experience can decrease the likelihood that someone would return for treatment during a future suicide crisis,^15^ potentially increasing the risk of suicide. However, many hospital staff report they do not have adequate time, training, or access to resources to provide the therapeutic care that individuals in suicidal distress need.^16,17^ Offering digital therapeutics in hospitals has the potential to cost-effectively improve or augment workforce capacity to provide evidence-based psychological care, without incurring additional substantial investment from an already strained hospital service.^18^

The strategic and operational complexities of hospitals mean that ‘off-the-shelf’ digital therapeutics may not appropriately meet the unique needs of patients in suicidal distress, nor the implementation needs of staff. Given that innovations with low acceptability typically have low engagement, and in turn, reduced clinical benefits,^19^ identifying design considerations relevant to hospitals is important to advancing implementation of digital therapeutics in these tertiary healthcare settings.

The engagement challenges posed by digital therapeutics^19,20^ have led to recent growth in ‘design thinking’ research which aims to identify enablers and barriers of use. In the field of suicide prevention, much of this research to date has: explored digital therapeutic use in community and secondary mental health care settings, focused on adult populations, and has been conducted in the context of understanding design issues relevant to already existing digital interventions (e.g. BlueIce, MYPLAN).^21–25^ Many of these methodological considerations extend to the one study that has sought to develop and test two mobile apps for emergency department patients with suicide risk.^24^ While these studies provide useful insights into modifying and optimising *existing* products, the findings may not generalise in ways that are useful to the development of new interventions that are optimally designed from inception to be integrated into hospital-provided care. The limited ‘youth-specific focus’ of many prior studies also means that the design considerations for enhancing the acceptability and relevancy of digital therapeutics for young people who attend hospital for suicidal distress are unclear.

To address these gaps, the aim of the current study is to understand and identify ‘general’ design considerations that could support researchers and industry to develop digital therapeutics that are fit-for-purpose to implement in hospitals to augment care provision, and which could effectively support young people presenting in suicidal distress.

## Method

This qualitative study uses data collected as part of a larger study which explored enablers and challenges to the implementation of digital therapeutics in Australian hospitals from the perspectives of young people who have previously presented to hospital for suicidal distress and the health professionals who care for them.^26^ The implementation findings have been reported elsewhere.^26^

### Study setting

Participants were recruited from across Australia via Black Dog Institute's website, Facebook page, and via monthly e-newsletters between May and November 2022. This study was approved by University of New South Wales Human Research Ethics Committee (HC210973), and informed, written consent was obtained by all participants prior to the interviews.

### Participants

Young people were eligible to participate in this study if they met the following criteria (via self-report):

1. Aged between 16 and 24 years (inclusive),
2. Were an Australian resident at the time of the study,
3. Had access to a computer or smartphone with internet connection,
4. Had presented to an Australian hospital for a suicidal crisis (ideation or self-harm with suicidal intent) in the past 12 months, and
5. Were able to speak and understand English fluently.

Participants were ineligible if they were flagged as having an active suicide risk as per the 3-item Patient Safety Screener (PSS-3).^27^ If an individual was deemed ineligible due to active suicide risk, as identified by endorsement of thoughts of suicide in previous 2 weeks and/or a suicide attempt in the past month, they were contacted within 48 h with an invitation for them to speak with a psychologist on the research team to ensure their safety. Young people aged 16 and 17 years were required to complete a brief, five-question Gillick Competency Assessment^28^ to determine if they understood what they were consenting to. All five questions needed to be answered correctly before an interview could be scheduled.

Hospital staff were eligible for participation if they were:

1. Currently employed (full- or part-time) in an Australian hospital (public or private),
2. Working in an administrative or clinical role during which they interact with young people experiencing mental health issues,
3. Had access to a computer or smartphone with internet connection, and
4. Aged 18 years or older.

There were no exclusion criteria for hospital staff.

### Data collection

Interview guides were developed in alignment with three of the factors of normalisation process theory (NPT)^29^: coherence – how people make sense of the intervention in practice; cognitive participation – how people engagement with new practices; and collective action – how new interventions become part of routine practice. NPT focuses on understanding how stakeholders (young people and hospital staff) make sense of new interventions as part of an implementation framework. No questions in the interview guide (see S1_Interview Guide) specifically asked about design or content considerations, instead the participants voluntarily provided this information in response to other questions about digital therapeutic appropriateness and integration.

All interviews were conducted online via a secure videoconferencing platform. Interviews with young people were conducted between May and October 2022; hospital staff were interviewed in November and December 2022. Interviews with each group were continued until saturation had been achieved. Participants were given a $50 (AUD) gift card as reimbursement. The initial four interviews with young people were conducted between two members of the research team (DR, and LM or MT), with the lead author (DR) conducting the remaining interviews (n = 25). Interviews were transcribed and any identifying information was anonymised.

### Data analysis

Data was analysed using Framework Analysis^30,31^ and Thematic Analysis^32^ approaches. Framework Analysis was utilised in alignment with the methodology of the larger study;^26^ however, it became clear that we had a wealth of data relating to the content and design of digital therapeutics which were outside the scope of the parent study aims. Data relating to content and design was separated out and analysed using Thematic Analysis. Data was managed via Nvivo Software (Version 20).

Initially analysis followed the first four steps of Framework Analysis and involved DR and RB familiarising themselves with the transcripts, then the first five transcripts from each participant group were coded independently, after which an analytic framework was developed by RB and DR which mapped the codes to NPT factors. This was then repeated with the remaining transcripts. Once codes were identified by thorough interrogation by DR, RB, and MT to be in relation specifically to the digital therapeutic content and design the codes were separated from the larger data set. Thematic analysis was conducted on the raw codes (sans the analytic framework) and involved DR, RB, and MT examining the codes for similarities and developing themes which consolidated overarching ideas. The final themes and codes were then synthesised for reporting.

## Results

### Participants

Interview data was collected from 17 young people (mean age: 18.4 years, SD: 2.9 years age range: 16–24 years), and 12 hospital staff (mean age: 35.4 years, SD: 11.6, age range: 21–56 years). The majority of the young people were female (*n *= 11, 65%), followed by non-binary (*n *= 4, 24%), then male (*n *= 2, 12%). The majority of hospital staff were female (*n *= 10, 83%) with only two males participating (17%). Most hospital staff worked as nurses (*n *= 5, 42%), followed by mental health clinicians (*n *= 3, 25%), peer support workers (*n *= 2, 17%), a mental health support worker (*n *= 1, 8%), and a hospital physician (*n* = 1, 8%). The majority of young people attended hospitals in metropolitan areas (*n *= 11, 65%), while majority of hospital staff worked in regional areas (*n *= 8, 67%).

The majority of young people had previously used a digital therapeutic in some capacity, not necessarily in a healthcare setting (*n* = 13, 76%), while less than half of hospital staff had used, or recommended, a digital therapeutic to a young person in suicide crisis or with mental health concerns as part of their provision of care (*n* = 7, 58%).

### Thematic analysis

Thematic analysis resulted in three themes which outlined the specific content needs for digital therapeutics when being utilised in a hospital setting: hospital-specific content, therapeutic content, and usability (see Table 1).

**Table 1.** Themes and codes within each, along with indication of young person and/or hospital staff endorsement.

Theme	Code	Reported by young person	Reported by hospital staff
Hospital-specific content	Information about what to expect		X
	Platform to consolidate information to staff	X	X
	Check-in function post-discharge	X	X
Therapeutic content	Coping strategies	X	X
	Distraction	X	X
	Self-reflection	X	X
	Safety planning		X
	Chat function	X	
Usability	Bespoke and customisable	X	X
	Inclusive design and language		X
	Easy to navigate	X	
	Engaging content	X	
	Continued access after hospital discharge	X	

Theme 1: Hospital-specific content. Hospital staff and young people identified content which was specific to improving the experience of providing, or receiving, care in the hospital. This included providing the young person with information about what they can expect from the hospital visit, the ability for staff to access the young people's responses to facilitate assessment and treatment planning, and finally, a mechanism to facilitate checking-in with the young person after they are discharged from the hospital.

### Information about what to expect

Staff saw a digital therapeutic as an opportunity to empower young people to navigate their care and their own needs, through an opportunity to inform them about the nature of the hospital care or community care journey. The community care journey refers to the steps planned for the young person's treatment as they transition from the hospital back to the community.

‘*If the young person in emergency department, it can be very daunting and if the apps can have some … really generic information* [about] *what might happen at the emergency department, then it gives them more relief so they know what is going on …’ – Hospital staff, Mental Health Clinician*

### Platform to consolidate information to staff

Hospital staff felt that having functionality within a digital therapeutic which linked patient self-report responses about their current suicide crisis with their clinical hospital records would help the patient begin to reflect and provide them an opportunity to understand the patient more prior to the assessment. Young people agreed with this and saw an option for staff to view their responses as a possible avenue to facilitate conversation and limit the volume which needed to be shared with staff verbally. Young people felt sharing information with staff would reduce feelings of loneliness. Hospital staff felt young people might be more comfortable disclosing sensitive information via a digital therapeutic rather than face-to-face, and as such this was a useful tool to facilitate a thorough risk assessment.

‘*I feel like it would be good. I feel like if they can link it in with hospital mental health teams as well, they can see who's using it, what they need help with, how they're feeling, everything like that. Then I feel like it definitely would be a lot more beneficial, and it would get rid of that lonely feeling. Yeah, I feel like it would be pretty good.’ – Young Person, 17 years*

‘*I definitely think it would open up a lot of doors that potentially are currently closed, if that makes sense. In a way that questions you ask as a clinician you feel a little bit, not uncomfortable, but unsure how to ask the questions in a respectful way. So potentially it allows the young people to get their feelings out and then as a clinician you can read that and approach questions in a way that are suitable, appropriate. … Or potentially young people might want to speak specifically about something but don't know how to start the conversation is probably another one as well. I guess it gives you a pathway into various different things that you can't get to as quick if you have that conversation, if that makes sense.’ – Hospital Staff, Mental Health Clinician*

### Check-in function post-discharge

Young people and hospital staff identified that a digital therapeutic may provide a preferred alternative to conduct the post-discharge check-in via the digital therapeutic rather than the typical phone call. Check-in via a digital therapeutic was perceived as less intrusive than a phone call.

‘*Instead of phone calls that last five minutes, it'd be good to just check in on an app and be like, “Yes. I'm doing great. I'm doing awful.” And then they can use that data maybe to help with your next follow-up, instead of a five-minute, “Are you alive?” phone call that I got.’ – Young Person, 17 years*

‘*Or even maybe being able to send a text from message media and saying, “Hey, just following up. Did you want to have a chat or are things all good?” That way, the person isn't put on the spot, and as a clinician, we don't feel like we're suggestive selling and saying, “Hey, how's your suicidality today?”’ – Hospital Staff, Nurse*

However, some hospital staff felt that while a check-in function post-discharge may be functionally better for young people, it did present other issues around who and how to respond to a suicide risk discloser if that were to happen during check-in.

‘*My concerns with it would be if there was anything disclosed to a concern. … Whereas when you do have that conversation face to face … you know they're in a safe space. So yeah, I think follow ups great, a hundred percent, just has to be monitored if that's a possibility.’ – Hospital Staff, Mental Health Clinician*

Theme 2: Therapeutic content. Young people and hospital staff suggested a variety of therapeutic content which could make a digital therapeutic more beneficial for young people in a suicide crisis. Content options included coping strategies, activities for distraction and self-reflection, guided safety planning, and the mechanism to start a chat with someone via the digital therapeutic.

### Coping strategies

Hospital staff and young people felt that a digital therapeutic would provide a good opportunity to educate young people about possible coping strategies to either reduce their current level of distress or to help them avoid distress in the future. This includes cognitive behavioural therapy, dialectical behaviour therapy, mindfulness, breathing techniques, distress tolerance, or ‘coping mechanisms’ more broadly. Young people and hospital staff felt coping strategies would be helpful to manage distress during long waiting times in the emergency department. Hospital staff elaborated that young people could continue to use coping strategies to improve their distress tolerance beyond the hospital visit.

‘*Like for one passing the time, but also going through a digital therapeutic. Like if you're going to be spending all that time in hospital and you can only see the emergency therapist for half an hour, you might as well be working on coping mechanisms, even if only one of them absorbs into it like into your brain.’ – Young Person, 22 years*

‘*I think it's so important for them to be able to learn those coping skills and distress tolerance and things like that because then,* [it] *gives them a way just to manage it instead of having to go and go to an ED (emergency department) where things can be rather uncomfortable. Just teaching them those skills.’ – Hospital Staff, Nurse*

### Distraction

Young people believed a digital therapeutic may offer them a distraction which would stop them focusing on the thoughts and feelings which are exacerbating their suicide crisis or reduce the negative impacts of the harsh emergency department environment.

‘*Yeah, like if I was to recommend any app for the ED (emergency department) it would be [one that] takes like a few hours to work through because of how much content it has … lots of mindfulness things … do you need a distraction? Pop some bubbles. Like that's helpful.’ – Young Person, 22 years*

Hospital staff saw this as an opportunity to alleviate the distress which would improve the quality of their assessments, improving patient flow through the hospital, and hopefully led to better care plans to improve mental health long term.

‘*… activities as a distraction to help regulate, you're getting that client … When you are seeing that client, at least you're getting a regulated one that's able to focus and talk about what's happening.’ – Hospital Staff, Mental Health Clinician*

‘*I do think that there is a role for it because at the moment we leave them alone with their thoughts for hours and hours and that is also not helpful and that doesn't help them get safe and that doesn't help them find a solution to their problems either. In fact, a lot of times it causes further detriment.’ – Hospital Staff, Doctor*

### Self-reflection

Hospital staff and young people saw benefit in content that focused on the young person reflecting on their mental state. Giving young people space to debrief, consider, and reflect on their situation at their own pace was seen as a benefit to assessment and a way to facilitate crisis management.

‘*I think, good to think more about having a sort of feedback for kind of writing your own feelings or where you're at. Whether it be a sliding scale of some sort which you can add notes to, some sort of system so you can do it in a simple way or you can write things down to express to the nurse when they come to do your observations or ask you how you're going while you're waiting for, whether it be a bed or another review or whatever in ED (emergency department).’ – Young Person, 19 years*

‘*So I think just having a space where the* [young person] *can think and maybe look, the young person can just look and get out what they're feeling would be helpful as well, away from that kind of really intense space.’ – Hospital Staff, Mental Health Clinician*

### Safety planning

Hospital staff saw an opportunity to digitise safety planning via an app – to improve the acceptability of the plan through joint agreement between staff, young person, and carers and to improved accessibility due to accessibility of devices.

‘*If the* [digital therapeutic] *can allow a young person and a parent to safety plan together, say what can we do before we hit red? That type of thing. Whether that's safety planning on separate phones that come together, I don't know. But allowing that connection to be there in a crisis but also not be there to allow, seek support from each other.’ – Hospital Staff, Mental Health Clinician*

### Chat function

Young people mentioned the desire for a chat function, either with a real person or a chat bot would be beneficial as the experience of a suicide crisis is often isolating, and they felt they would benefit from interpersonal connection (even if artificial).

‘*I think the whole base around people being suicidal and depressed and giving up is because they think no one cares about them. So, I think having even someone you don’t know that's like a stranger, taking an interest and talking to you and wanting to find out what the problem is. It's sort of like more comforting I think’ – Young Person, 16 years*

Theme 3: Usability. Digital therapeutic content also needs to focus on how easy and enjoyable it is for the young person to engage with it. Hospital staff and young people both noted that a digital therapeutic which is personalised is ideal; hospital staff also highlighted the importance of inclusivity in design and language, and young people wanted the content to be easy to navigate, with the option to continue to access the digital therapeutic once discharged from the hospital.

### Bespoke and customisable

Hospital staff and young people commented that one of the limitations of existing digital therapeutics is that they lack the ability to be tailored to the users’ specific needs. Furthermore, participants identified an option to personalise the digital therapeutic's look and feel would increase their engagement.

‘*I like being able to personalise stuff. So, it would be like, “Hi* [name]*, welcome back,” and stuff like that. I don't know if that's just me, but it kind of makes it feel like my own space if I can choose colours and all that stuff, which is super simple, but having the basic stuff, like meditation or strategies or online therapy links and stuff. But just having the ability to personalise it and stuff would make it, I don't know, just feel more for me.’ – Young Person, 17 years*

‘*But if you had some way of going, “Oh, do you feel like this?” And then it takes you down this avenue … so you are homing in on a specific part of what's wrong. So, is it anxiety related or is it situational related? Is it something that just needs something to distract them or does it need fixing …’ – Hospital Staff, Nurse*

### Inclusive design and language

Hospital staff identified that a digital therapeutic should be inclusive and accessible to all people regardless of socio-demographic background, access to a device, abilities, neurodivergence, different ethnicities, and languages, among others. Staff also highlighted the importance of inclusive language and such as the user selecting their own gender identification.

‘*Because you've got neurodiversities to consider. You've got disabilities to consider. They may not be able to read and write. They might have the motor skills to use an app or is it functional for carers or family members to fill in for them? They're disabled, they may have a carer … the other bit I would ask a favour too, just because I'm an advocate in this space is LGBT stuff. Like include flags, include pronouns, preferred names, all of this stuff, non-binary sexes, all that stuff’ – Hospital Staff, Peer Support Worker*

### Easy to navigate

Young people indicated that any digital therapeutic being utilised for suicide crisis should be straightforward to use and have a simple navigation tutorial built into the beginning. Young people said it was difficult for them to concentrate on external things during a suicide crisis so having a digital therapeutic which is easy to navigate is important.

‘*I feel like most apps already have a “this is how you use the app” and it kind of comes up as a pop up when you just download it and then it disappears.’ – Young person, 18 years*

‘*I think it would have to be well designed, and well laid out, and run quite smoothly. Because a lot of mental health apps are quite clunky, and you can tell that they've been made with not a very big budget. Whereas Headspace, which you pay for, is beautifully designed, and everything is clear, because you have to pay to get it. But I think that's the thing, people will stop using it straight away if it's not a nicely designed app that's really simple to use and nice to look at as well.’ – Young Person, 23 years*

### Engaging content

Young people reflected that digital therapeutics they have utilised in the past were too generic and did not always provide unique and helpful information. Young people want digital therapeutic content, which is engaging and interesting, so they could get the most benefit from it. Some suggestions included novel mental health education and strategies, providing lots of different activities to engage with, and positive stories of other people overcoming their crisis.

‘*I know people who they actually really help with and the meditation part of it, that helps them sleep and those little five-minute brain puzzle things, they help them.’ – Young Person, 17 years*

‘*Probably the option to hear real life stories of how people have turned their life around or what's helped other people get through a suicide attempt or real-life testimonials like videos and stuff.’ – Young Person, 24 years*

### Continued access after hospital discharge

Some young people felt that being able to continue to use the app after leaving the hospital would be beneficial. This would help them to continue to benefit from the hospital visit and provide them tools to continue to manage their suicide crisis when in the community. Young people also felt that they would likely benefit more from a digital therapeutic if being used outside of the hospital as well, since they are likely out of an immediate crisis and more receptive to information.

‘*I think it would be helpful for after you've gotten at the hospital as well, like if you've already worked for it, and then you have it on your phone, like you, more likely to open it after.’ – Young Person, 22 years*

## Discussion

This study qualitatively explored young people and hospital staff's content and design recommendations for digital interventions for young people in suicidal distress, as part of broader implementation considerations in hospital settings. Participants provided recommendations for content which would benefit either the young person or the hospital staff in a hospital setting, therapeutic content which is appropriate during suicidal distress, and design considerations which enhance the usability of a digital therapeutic for young people in suicidal distress.

A digital therapeutic is more likely to become embedded into routine practices in the hospital and as part of community-based aftercare if it benefits both young people and hospital staff.^33^ Both young people and hospital staff identified the benefit of being able to consolidate the information the young person shares in the digital therapeutic with the hospital records. Hospital staff, particularly those in the emergency department, have limited time to speak with a patient,^17,34^ which can impact their ability to build rapport or get a sound understanding of an individual's suicide presentation.^17^ In this study, participants identified that linking the digital therapeutic with hospital data could facilitate rapport building and allow staff to efficiently understand a young person's situation so they can focus on their specific needs. Young people have reported the discomfort in having to recall their suicidal crisis numerous times as they tell multiple staff members.^13^ The ability to provide this information digitally, which all staff could read before speaking with the young person, could limit the repetitive story telling required of young people in suicidal crisis, and alleviate some of the distress due to limited privacy in the hospital setting.^13^ Moreover, as prior research has shown that using both data from patient self-report *and* clinician assessments together can improve clinical risk assessment accuracy,^35^ being able to capture the proximal circumstances surrounding a young persons’ suicidal crisis could add value to clinicians in respect to risk assessment and improved care planning and management.

There was strong agreement between young people and hospital staff that digital therapeutics should support coping skill development both inside and outside the hospital. Long periods of waiting are commonplace in emergency departments^13,14,36,37^ and this was identified as ‘wasted time’ by health professionals in our study, who saw value in using this time to use digital tools to teach young people new coping strategies. This complements prior research showing that young people want, and benefit from, digital therapeutics that teach adaptive coping strategies,^22,23,38^ and that these strategies continue to be important in the post-discharge period.^39^ Mental health professionals working within the emergency department are often unable to provide therapeutic intervention, instead focusing on assessing risk and determining future directions for care.^40^ Hospital staff recognised that helping young people develop coping skills was a key gap in their care delivery. Young people identified that therapeutic modalities such as mindfulness and cognitive behavioural therapy could be beneficial during time of distress; however, they also wanted information about how to manage the stressful or overwhelming instances they may have to deal with in the future, to hopefully decrease their risk of experiencing in suicidal distress in the future.

The findings suggested that personalisation functionality was important to young people, including in the visual design (e.g. colour schemes) and in being able to ‘favourite’ strategies or information that particularly appealed to them, as it would foster a sense of ownership and empowerment which in turn may increase engagement with these tools. The preference for similar ‘personalisation’ strategies has emerged as an ‘enabling’ design consideration in other comparable studies,^22,23^ suggesting this is a valued feature that generalises across healthcare settings and intervention types, and may offer a cost-effective opportunity to meet users’ needs. Almost a quarter of young people identified as non-binary (24%) and hospital staff highlighted the importance of the digital therapeutic tool having an option to specify gender-identify, where relevant, to promote inclusion. The tool also needs to be available in a variety of languages to cater to the needs of young people in multicultural Australia. A recent scoping review found that only 58% of mental health app evaluation frames included *any* considerations for divesity, equality, and inclusion.^41^

Some important implications emerge from this study. While new digital therapeutics are being rapidly integrated into hospital settings to support individuals in suicide crisis over recent years,^42–45^ none have specifically targeted young people. This study provides novel insights into the design preferences of young people that can inform the design of setting-specific interventions that are optimised for engagement. It, however, remains to be established which design considerations are practical to implement and would actually improve care provision in hospitals. While this study explored design considerations specifically for a hospital setting, future studies should consider exploring how digital therapeutics may be designed to facilitate coordinated care across the different tiers of the healthcare system (i.e. primary, secondary, and tertiary), as using digital tools to support ‘care continuity’ may improve outcomes for individuals experiencing suicidal thoughts and behaviours.^46^ Not all design considerations were consistently raise by both young people *and* hospital staff. Additional research is needed to interrogate the design considerations where perspectives diverged between these groups (e.g. the use of chat bots, the need for inclusive language). Understanding what design features are meaningful for whom, and in what circumstances, is important for designing tools that optimise effectiveness and value-based care. Further to this point, it would be useful to unpack what the ‘active treatment ingredients’ of digital tools are, specifically those that are robustly linked to modifications in suicidal thoughts and behaviours. Such insights could help inform design thinking as to how these ‘ingredients’ should be delivered in high-pressure settings, such as hospitals, to support young people.

There are several limitations to consider. First, the majority of staff had not utilised a digital therapeutic before in the care of individuals or for their personal use. This may make it difficult for them to conceptualise what content and design features would be the most beneficial for themselves and young people. Second, the larger proportion of female participants may have influenced the type of content and design considerations provided. Third, staff were from a variety of hospitals across Australia, limiting the ability to make assumptions about what would work within specific hospitals or local health areas. Similarly, there were no intentional or obvious young people-hospital staff dyads, making it difficult to draw conclusions about shared experiences. Despite these limitations, there was considerable agreement between the two groups, suggesting that the recommendations made by the two groups may be broadly applicable.

## Conclusion

This study advances current understandings of digital therapeutics specific to the hospital setting. The results suggest that there are unique design and functionality considerations specific to hospital settings if digital therapeutics are to effectively improve the provision of care for young people in self-harm and suicide distress. These design considerations could inform the development of new interventions or augment existing ones to ensure they are appropriately retrofitted to the needs of complex, high-volume care settings.

## Supplemental Material

sj-docx-1-dhj-10.1177_20552076241230072 - Supplemental material for Digital therapeutics in the hospital for suicide crisis – content and design recommendations from young people and hospital staff Click here for additional data file. Supplemental material, sj-docx-1-dhj-10.1177_20552076241230072 for Digital therapeutics in the hospital for suicide crisis – content and design recommendations from young people and hospital staff by Demee Rheinberger, Rachel Baffsky, Lauren McGillivray, Daniel Z Q Gan, Mark Larsen and Michelle Torok in DIGITAL HEALTH
