# Supplementary material for: Digital therapeutics in the hospital for suicide crisis – content and design recommendations from young people and hospital staff
Source: Digit Health. 2024 Feb 14;10:20552076241230072. doi: 10.1177/20552076241230072 (PMC10868481; doi:10.1177/20552076241230072)
Supplement: sj-docx-1-dhj-10.1177_20552076241230072 - Supplemental material for Digital therapeutics in the hospital for suicide crisis – content and design recommendations from young people and hospital staff [file sj-docx-1-dhj-10.1177_20552076241230072.docx]

S1_Checklist: COREQ

| **Domain 1: Research team and reflexivity** | **Explanation** | **Location in manuscript**  **(Section, page #)** |
| --- | --- | --- |
| **Personal Characteristics** |  |  |
| 1. Interviewer/facilitator Which author/s conducted the interview or focus group? |  | Methods, pg 4 |
| 2. Credentials  What were the researcher’s credentials? E.g. PhD, MD |  | Title page |
| 3. Occupation  What was their occupation at the time of the study? | Mental health researchers | - |
| 4. Gender Was the researcher male or female? | Female | - |
| 5. Experience and training  What experience or training did the researcher have? | All researchers had extensive experience engaging in research in relation to suicide prevention, digital therapeutics and/or qualitative methods | - |
| **Relationship with participants** |  |  |
| 6. Relationship established  Was a relationship established prior to study commencement? | No | - |
| 7. Participant knowledge of the interviewer What did the participants know about the  researcher? e.g. personal goals, reasons for doing the research | Participants were briefed on the purpose of the study, that ethical approval had been granted, participants reviewed the participant information documentation prior to giving their written informed consent to  be involved. All participants had the opportunity to ask any questions prior to interview commencement | - |
| 8. Interviewer characteristics What characteristics were reported about the interviewer/facilitator? e.g. Bias, assumptions, reasons and interests in the research topic | No | - |
| **Domain 2: study design** |  |  |
| **Theoretical framework** |  |  |
| 9. Methodological orientation and Theory What methodological orientation was stated to  underpin the study? e.g. grounded theory, discourse analysis, ethnography, phenomenology, content analysis | Thematic analysis, normalization process theory | Methods – pg 4 |
| Participant selection |  |  |
| 10. Sampling How were participants selected? e.g. purposive, convenience, consecutive, snowball | Recruited via social media and newsletters | Methods – pg 3 |

| 11. Method of approach How were participants approached? e.g. face-to-face, telephone, mail, email | Email |  |
| --- | --- | --- |
| 12. Sample size How many participants were in the study? | 17 young people and 12 hospital staff | Results – pg 4 |
| 13. Non-participation How many people refused to participate or dropped out? Reasons? | 8 young people and 2 hospital staff opted not to participate in the study prior to the interviews.  No participants withdrew after data collection. | - |
| **Setting** |  |  |
| 14. Setting of data collection  Where was the data collected? e.g. home, clinic, workplace | Data was collected via online interviews via secure videoconferencing platform | Methods – pg 4 |
| 15. Presence of non-participants  Was anyone else present besides the participants and researchers? | No | - |
| 16. Description of sample  What are the important characteristics of the sample? e.g. demographic data, date | Young people mean age: 18.4 years, 65% female. Hospital staff mean age: 35.4 years, 83% female, majority working as nurses 42%.  Data was collected between May and December 2022 | Results – pg 4-5 |
| **Data collection** |  |  |
| 17. Interview guide Were questions, prompts, guides provided by the authors? Was it pilot tested? | Interviews were semi-structured, interview guides attached (Appendix A). The interview guide was tested on the first 4 participants to ensure suitability. | Methods – pg 4 |

| 18. Repeat interviews  Were repeat interviews carried out? If yes, how many? | No | - |
| --- | --- | --- |
| 19. Audio/visual recording  Did the research use audio or visual recording to collect the data? | The semi-structured interviews were audio recorded via the video conferencing software. | - |
| 20. Field notes  Were field notes made during and/or after the interview or focus group? | No additional field notes were made. | - |
| 21. Duration What was the duration of the interviews or focus group? | The semi-structured interview durations ranged from approximately 30 to 90 minutes | - |
| 22. Data saturation  Was data saturation discussed? | Yes | Methods – pg 4 |
| 23. Transcripts returned Were transcripts returned to participants for comment and/or correction? | No | - |
| **Domain 3: analysis and findings** |  |  |
| **Data analysis** |  |  |
| 24. Number of data coders  How many data coders coded the data? | Two | Methods – pg 4 |
| 25. Description of the coding tree  Did authors provide a description of the coding tree? |  | Methods – pg 4 |
| 26. Derivation of themes  Were themes identified in advance or derived from the data? | Themes were derived from the data | Methods – pg 4 |
| 27. Software What software, if applicable, was used to manage the data? | Nvivo Software (version 14) | Methods - pg 4 |
| 28. Participant checking  Did participants provide feedback on the findings? | No | - |
| **Reporting** |  |  |
| 29. Quotations presented Were participant quotations presented to illustrate the themes / findings? Was each  quotation identified? e.g. participant number | Yes, specific comments were supported with direct quotes attributed to anonymized participant by gender and age (young people) or profession (hospital staff) | Results – pg 6-10 |
| 30. Data and findings consistent  Was there consistency between the data presented and the findings? | Yes | Results – pg 6-10 |
| 31. Clarity of major themes  Were major themes clearly presented in the findings? | Yes | Results –pg 6-10 |
| 32. Clarity of minor themes  Is there a description of diverse cases or discussion of minor themes? | Yes | Results – pg 6-10 |

# S1 Interview Guide

# Interview questions for young people

Questions about experience presenting to hospital for a suicidal crisis.

- When did you last present to a hospital for a suicidal crisis – that is, either suicidal thoughts, plans, or self-harm behaviour?
  - Was this your first presentation for a suicidal crisis?
  - If no, tell them “The next set of questions will just relate to the most recent presentation if that’s okay.”
- What kind of care did you receive in hospital for a suicidal crisis?
  - Would you consider this care appropriate for what you presented with?
  - How organized do you feel the care was?
- Did you have contact with one key person throughout your care, or were lots of different staff involved?
- What was the role of the staff that you mainly interacted with while in hospital (e.g. psychologist, ED nurses, nurse admin)
  - What was that like (either having the same person or lots of different people involved)?
- Were care options communicated to you? (e.g. …….)
  - If yes, what were they?
  - If yes, when were care options communicated to you?
  - If yes, who communicated them – what was their job role?
- If they presented to hospital more than once, ask “you said that you had presented to hospital more than once – can you tell me if the care you received change at different visits and how?”

For the next set of questions, we’ll focus more on digital therapeutics and how they might have fit into your hospital experience.

- Do you have any experience with digital therapeutics or know anyone who has?
- What do you think about digital therapeutics – why do you say this?
- Can you think of any advantages or disadvantages of digital therapeutics?
  - Do you think there is any added value of digital therapeutics to help manage suicidal crises – like, what could they offer, that face-to-face or telehealth can’t?
- Do you think digital therapeutics apps require knowledge or skills that are different to other apps?
  - If so, what?
  - Which of these skills and knowledge do you already have?
  - What would be the best ways to help you to develop these skills and this knowledge?
- How do you feel about digital therapeutics being offered as a care option in hospital? Why do you feel that way?
- If they were offered to you at hospital, at what stage do you think they should be offered? (e.g. should they be offered while waiting in the ED, upon discharge from an ED, as part of in-patient mental health services, or not at all)?
- How would you prefer to use a digital therapeutic – by yourself, with clinician support, with a family member or friend, part of face-to-face therapy, or perhaps another way – why do you say this?
- What would help you to use digital therapeutics – why do you say this?
- Is there anything that might prevent you from using digital therapeutics?

# Interview guide for the hospital staff participant group

I might start by asking you about your role and experience providing care to young people in a suicide crisis.

- Can I ask you what your profession is? (title, ED/ward/hospital, metro/regional/rural)
- What does your role look like – specifically in relation to the care of young people in suicide crisis?
- How long have you been involved in the care of young people in suicide crisis?
- How regularly are you engaging with young people in a suicide crisis?
- Can you briefly explain what care young people typically receive from your hospital when they present for a suicide crisis?

### Questions about digital therapeutics

- What is your experience with digital therapeutics?
- What do you think are the key advantages or disadvantages of digital therapeutics for supporting young people in crisis?
- In what ways could digital therapeutics add value for how you currently support young people? Could they address gaps in care?
- Do you think digital therapeutics could be an appropriate care option for young people who present to the hospital/ED during a suicide crisis?
- If a digital therapeutic was offered to young people in the hospital, what should be included in it?
- When do you think digital therapeutics should be offered to young people in suicide crisis? (eg in the waiting room, admitted into the ED, in in-patient facilities, on discharge)
- How should they be offered – for instance, who should be involved and what would their role be?
  - Do you think follow up on digital therapeutic use would be beneficial?
- Do you feel like an appropriate person to inform a young person about digital therapeutics?
  - If so, why?
  - If not, who do you think should be offering it?
- What skills and knowledge would help a young person to benefit from digital therapeutics?
  - Which of these do you have?
  - What would be the best ways to help you or other hospital staff to develop these skills and this knowledge?
- Think about the times when the hospital underwent considerable change (e.g., introduction of a new practice or a different model of care) – what enabled that to happen?
- What hindered the change and how were these challenges managed?
- How easy do you think it would be to introduce digital therapeutics as a care option in hospitals?
- What would do you think would help or hinder the introduction of digital therapeutics in hospitals/your hospital?
- How receptive do you think your hospital would be to the introduction of digital therapeutics to manage suicidal crises – why do you say this?
- How could that receptiveness be enhanced – for instance, who should be involved and how?

“What further comments would you like to add? What did you expect us to ask or talk about, which wasn’t addressed? How could we have improved this discussion for you?”
